# Utility of the Rapid Antigen Detection Test E. histolytica Quik Chek for the Diagnosis of Entamoeba histolytica Infection in Nonendemic Situations

**DOI:** 10.1128/JCM.01991-20

**Published:** 2020-10-21

**Authors:** Yasuaki Yanagawa, Rieko Shimogawara, Tomoyuki Endo, Rika Fukushima, Hiroyuki Gatanaga, Kasumi Hayasaka, Yoshimi Kikuchi, Taiichiro Kobayashi, Michiko Koga, Tomohiko Koibuchi, Toshikazu Miyagawa, Ayaka Nagata, Hirotomo Nakata, Shinichi Oka, Risa Otsuka, Kazumi Sakai, Mami Shibuya, Hiroyuki Shingyochi, Etsuko Tsuchihashi, Koji Watanabe, Kenji Yagita

**Affiliations:** a AIDS Clinical Center, National Center for Global Health and Medicine, Tokyo, Japan; b Department of Parasitology, National Institutes of Infectious Diseases, Tokyo, Japan; c Department of Hematology, Hokkaido University Hospital, Hokkaido, Japan; d Division of Laboratory Diagnosis, Kumamoto University Hospital, Kumamoto, Japan; e Joint Research Center for Human Retrovirus Infection, Kumamoto University, Kumamoto, Japan; f Division of Laboratory and Transfusion Medicine, Hokkaido University Hospital, Hokkaido, Japan; g Department of Infectious Diseases, Tokyo Metropolitan Cancer and Infectious Diseases Center Komagome Hospital, Tokyo, Japan; h Research Hospital Division of Infectious Diseases and Applied Immunology, The Institute of Medical Science, The University of Tokyo, Tokyo, Japan; i Department of Hematology, Rheumatology, and Infectious Diseases, Kumamoto University, Kumamoto, Japan; j Laboratory Testing Department, National Center for Global Health and Medicine, Tokyo, Japan; k Department of Clinical Laboratory, Tokyo Metropolitan Cancer and Infectious Diseases Center Komagome Hospital, Tokyo, Japan; l Research Hospital Division of Laboratory Diagnosis, The Institute of Medical Science, The University of Tokyo, Tokyo, Japan; Mayo Clinic

**Keywords:** parasitology, amebiasis, diagnosis, cyst, *Entamoeba histolytica*, parasitology

## Abstract

Entamoeba histolytica infection is an increasingly common sexually transmitted infection in Japan. Currently, stool ova and parasite examination (O&P) is the only approved diagnostic method. Here, we assessed the utility of the commercially available rapid antigen detection test (Quik Chek) for E. histolytica. A multicenter cross-sectional study was conducted. Stool samples that had been submitted for O&P were included. The samples were subjected to both Quik Chek and PCR, and the Quik Chek results were assessed in comparison with PCR as the reference standard. E. histolytica infection was confirmed in 5.8% (38/657) of the samples and comprised 20 diarrheal and 18 nondiarrheal cases. The overall sensitivity and specificity of Quik Chek were 44.7% (95% confidence interval, 30.1 to 60.3) and 99.8% (99.1 to 100), respectively. The sensitivity of Quik Chek was higher for diarrheal cases (60.0%) than for nondiarrheal cases (27.8%). Furthermore, the combined use of Quik Chek with O&P increased the sensitivity (78.9%), especially for diarrheal cases (up to 90%). The E. histolytica burden assessed by quantitative PCR was similar between Quik Chek-positive and -negative samples. The Quik Chek assay sensitivity was lower for cyst-containing stools than for trophozoite-containing stools, although it was shown that cultured E. histolytica clinical strains from Quik Chek-negative cyst-containing stools exhibited antigenicity *in vitro*. The present study confirmed the high specificity of Quik Chek for E. histolytica infection. Combined use with O&P increased the sensitivity of detection, facilitating the use of Quik Chek in point-of-care settings in nonendemic situations.

## Abstract (precis)

Entamoeba histolytica infection is an increasingly common sexually transmitted infection in Japan. Currently, stool ova and parasite examination (O&P) is the only approved diagnostic method. Here, we assessed the utility of the commercially available rapid antigen detection test (Quik Chek) for E. histolytica. A multicenter cross-sectional study was conducted. Stool samples that had been submitted for O&P were included. The samples were subjected to both Quik Chek and PCR, and the Quik Chek results were assessed in comparison with PCR as the reference standard.

## INTRODUCTION

Entamoeba histolytica, the causative agent of invasive amebiasis, is the second most common parasitic cause of mortality worldwide (1). Over the past 2 decades, invasive amebiasis has become prevalent not only in developing countries, where food and water are frequently contaminated by feces, but also in several developed countries in Asia and Europe (2–5). In these areas, the pathogen spreads as a sexually transmitted infection, especially among men who have sex with men and within the HIV-infected population (2, 5, 6). Furthermore, recent data indicate that this pathogen is also spreading among HIV-uninfected men and women in Japan (7–9).

In Japan, stool ova and parasite examination (O&P) is the only approved diagnostic method for E. histolytica infection as of 25 August 2020. O&P is a low-cost and rapid diagnostic tool for enteric parasite infection; however, in most developed countries, it is not recommended for the diagnosis of E. histolytica (10, 11). This is because (i) O&P cannot distinguish nonpathogenic *Entamoeba* spp., such as E. dispar and E. moshkovskii, from potentially pathogenic E. histolytica, and (ii) the sensitivity and specificity of O&P are highly dependent on local health care settings, such as the skill of the technician and the timing of the examination, as O&P should be carried out by a well-trained laboratory technician immediately (within 1 h) after sampling. Moreover, enteric parasite infections are not common in regions with good hygiene, resulting in many developed countries having fewer opportunities for the training of health care professionals in the O&P technique. The PCR is the most reliable diagnostic tool for the detection of E. histolytica; however, this procedure, which includes the extraction of DNA from various types of clinical samples, remains too technically complex for point-of-care use and too expensive for the routine clinical diagnosis of E. histolytica infection (10, 11). On the other hand, E. histolytica antigen detection tests are easy to perform and relatively inexpensive. For example, the E. histolytica Quik Chek test is an FDA-approved rapid test based on immunochromatography, and the utility of this test has been proven in several studies performed in developing countries (12–14). However, reports from developed countries are limited (15).

In the present study, we assessed the utility of Quik Chek through a multicenter cross-sectional study in Japan, and we sought to determine the best use of this technique to improve the diagnosis of amebiasis in clinical settings.

## MATERIALS AND METHODS

### Study design and sampling.

This multicenter cross-sectional study was carried out between October 2018 and December 2019. Clinical specimens were prospectively collected from patients with suspected enteric parasite infections at five regional core hospitals in Japan, including the National Center for Global Health and Medicine (primary facility, Tokyo), Hokkaido University Hospital (Hokkaido), Tokyo Metropolitan Cancer and Infectious Diseases Center Komagome Hospital (Tokyo), The Institute of Medical Science at The University of Tokyo (Tokyo), and Kumamoto University Hospital (Kumamoto). All stool samples, which had been submitted for O&P for diagnostic purposes, were recruited for the present analysis after anonymization. Clinical information other than the O&P diagnosis and stool form as scored with the Bristol stool scale were not handled in the present study. Thereafter, the collected specimens were examined with the Quik Chek assay (E. histolytica Quik Chek; Techlab, Blacksburg, VA, USA) independently from O&P at each institution. The remainder of the specimens was stored at −20°C and transferred to the National Institute of Infectious Diseases (Tokyo) for PCR detection of E. histolytica and other protozoa. This study was approved by the ethics committee of each facility as well as the National Center for Global Health and Medicine Center (approval no. NCGM-G-002516-00). This study was implemented in accordance with the provisions of the Declaration of Helsinki.

### DNA extraction from stool samples.

Stool specimens (approximately 0.2 g) were weighed and subjected to DNA extraction using a QIAamp Fast DNA Stool minikit (Qiagen, Hilden, Germany). DNA extraction was performed according to the manufacturer’s instructions and a previous report (16). The DNA was eluted in 100 μl of elution buffer (Qiagen) and stored at −80°C until further analysis.

### Conventional and quantitative PCR.

A single-round conventional PCR (cPCR) assay for the detection of three *Entamoeba* species (E. histolytica, *E. dispar*, and *E. moshkovskii*) was carried out. The primer set was designed based on signature sequences in the small-subunit rRNA of each species, of which, the utility was confirmed in a previous study (17). The primer set consisted of the same forward primer (EntaF, 5′-ATGCACGAGAGCGAAAGCAT-3′) in combination with three reverse primers, one for each of the three species (EhR, 5′-GATCTAGAAACAATGCTTCTCT-3′; EdR, 5′-CACCACTTACTACC-3′; EmR, 5′-CACCACCACTTACTATCCCTACC-3′). *Entamoeba* species were differentiated based on the sizes of the PCR products (a 166-bp PCR product for E. histolytica, a 752-bp PCR product for *E. dispar*, and a 580-bp PCR product for *E. moshkovskii*). Finally, the results were confirmed by DNA sequencing. For cases in which E. histolytica infection was confirmed by cPCR, quantitative PCR (qPCR) was additionally performed using a 6-carboxyfluorescein (FAM)-conjugated probe (TCATT+GAATGAATTGGCCATTT) and an *Entamoeba* primer set (Ehd-88R, 5′-GCGGACGGCTCATTATAACA-3′, and EM-RT-F2, 5′-GTCCTCGATACTACCAAC-3′) (18). The pathogen burden of E. histolytica was presented as the quantity of trophozoite DNA per milligram stool relative to standard reference samples from an axenically cultured experimental strain (HM-1:IMSS).

### Antigen detection test using the E. histolytica Quik Chek assay.

For the antigen detection test, we used the E. histolytica Quik Chek (Techlab, Blacksburg, VA, USA) assay according to the package insert. In brief, all reagents and specimens were brought to room temperature before testing. Next, 25 μl of liquid stool or a 2-mm-diameter portion of solid stool homogenized with 500 μl of diluent was premixed with a drop of the conjugate. All of the diluted sample (∼500 μl) was added to a sample well of the test membrane in the device and incubated for 15 min. Finally, 300 μl of wash buffer, followed by two drops of the substrate, was added directly to the reaction window.

### E. histolytica culture from clinical specimens.

Cultivation of E. histolytica was attempted for all cases diagnosed as having E. histolytica infection at the National Center for Global Health and Medicine, within the ambit of the written informed consent obtained for another study (approval no. NCGM-G-001566-02). Isolation of axenic E. histolytica was performed in accordance with the protocols previously published by Clark and Diamond (19). In brief, stool samples were first inoculated into xenic culture media (e.g., BR medium [R medium precultured with Escherichia coli] followed by Robinson medium), either directly for trophozoite-containing diarrheal stools or after initial treatment with 0.1 N hydrochloric acid for 10 min for cyst-containing formed stools. Thereafter, E. histolytica was cultivated in the trophozoite form in xenic culture media for several weeks or months. After obtaining stable growth in xenic culture media, monoaxenic culture was achieved by washing xenically cultured E. histolytica in phosphate-buffered saline and placing it into a rich medium containing Crithidia fasciculata and antibiotics (e.g., 8,000 U penicillin G, 0.04 g streptomycin, 12,000 U polymyxin B, plus 0.2 ml of antibiotic antimycotic 100× solution [Sigma-Aldrich, Merck KGaA, Germany] in 4 ml of medium). After cultivation in the monoxenic medium for several weeks or months, E. histolytica was finally cultured in *Crithidia*-free medium. The axenic clinical strains were then maintained in YIMDHA-S medium.

### Statistical analyses.

In the present study, cases were defined as being positive for E. histolytica infection where the identification of E. histolytica was confirmed by PCR in stool samples. The sensitivity and specificity of O&P or the antigen detection test were calculated with reference to the PCR data. Comparisons of the qualitative data were carried out with the chi-square test, and analysis of variance (ANOVA) was used for comparisons of quantitative data. Statistical significance was defined as a two-sided *P* value of <0.05. All statistical analyses were performed using GraphPad Prism 7.0 (GraphPad Software, Inc., San Diego, CA, USA).

## RESULTS

### Study subjects.

**FIG 1:**
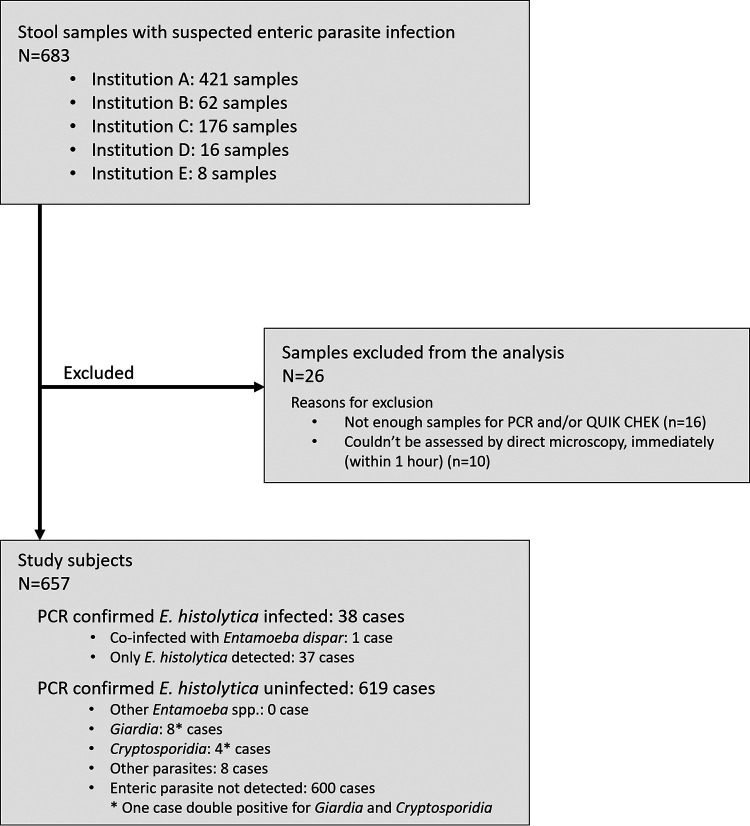
Workflow of the clinical specimen collection. Institutions A, B, C, D, and E are National Center for Global Health and Medicine (primary facility, Tokyo), The Institute of Medical Science at The University of Tokyo (Tokyo), Tokyo Metropolitan Cancer and Infectious Diseases Center at Komagome Hospital (Tokyo), Kumamoto University Hospital (Kumamoto), and Hokkaido University Hospital (Hokkaido), respectively.

**FIG 2:**
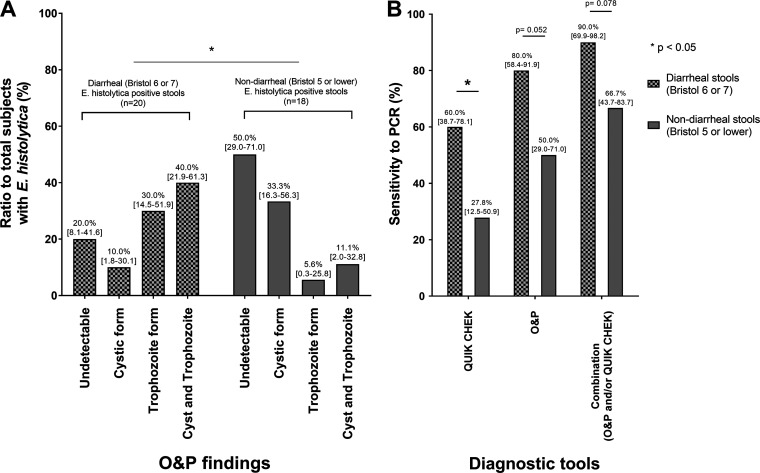
O&P findings and diagnostic sensitivities among diarrheal and nondiarrheal cases. (A) Morphologic diagnosis by stool ova and parasite examination (O&P) of the 657 samples included in this study identified 38 that were positive for E. histolytica. The number of cases in each subgroup of the O&P findings is expressed as a ratio to the total number of subjects in each of the diarrheal stool (*n* = 20) and formed stool (*n* = 18) groups. (B) The sensitivity of each diagnostic tool was compared between diarrheal and nondiarrheal stool samples. When using a combination of O&P and Quik Chek, a sample was deemed positive when either O&P or the antigen detection test was positive. O&P, ova and parasite examination; Quik Chek, rapid antigen detection test (E. histolytica Quik Chek).

In total, 683 stool samples were collected during the study period (Fig. 1; see also Fig. S1 in the supplemental material). Of these, 26 were excluded from the analyses, either because there was insufficient sample remaining after O&P (16 samples) or O&P could not be performed immediately after sampling due to the unavailability of a skilled laboratory technician (10 samples). Therefore, 657 samples were included for further analysis to assess the utility of Quik Chek. E. histolytica PCR identified 5.8% (38/657) of samples as positive, including 20 diarrheal cases (Bristol score 6 or 7) and 18 nondiarrheal cases (Bristol score 5 or lower). During O&P, trophozoite forms were observed more frequently in diarrheal stools, whereas the cystic forms were more frequently seen in formed stools (Fig. 2A). Coinfection with *E. dispar* was reported in one of the E. histolytica-positive diarrheal cases. On the other hand, among the 619 E. histolytica-negative samples, enteric parasites were detected in 19 cases, and these included eight cases of *Giardia* and four cases of *Cryptosporidium*. However, nonpathogenic *Entamoeba* spp. were not reported in the E. histolytica-negative samples, although recently discovered E. bangladeshi was not assessed in the present study (Fig. 1). These results suggest that E. histolytica infection is the most common enteric parasite in Japan, which emphasizes the need for well-constructed diagnostic systems for E. histolytica infection.

### Diagnostic value of E. histolytica antigen testing and O&P.

Next, we investigated the sensitivity and specificity of Quik Chek and O&P using the PCR results as a reference standard (Table 1). The sensitivity of Quik Chek was 44.7% (95% confidence interval, 30.1% to 60.3%), which was significantly lower than that of O&P (65.8% [49.9% to 78.8%]). On the other hand, the specificity of Quik Chek was 99.8% (99.1% to 100%). No cross-reactivity was seen with the other protozoa infections. Interestingly, the specificity of O&P was equivalent to that of Quik Chek in this study population, probably because nonpathogenic *Entamoeba* spp. are rarely seen in Japan. When a combination of O&P and Quik Chek was applied to the diagnosis of E. histolytica infection, and positive samples were defined as having either a positive O&P or Quik Chek result, the sensitivity and specificity were 78.9% (63.7% to 88.9%) and 99.7% (98.8% to 99.9%), respectively (Table 1). For the diarrheal cases, in particular, the combined use of O&P and Quik Chek increased the sensitivity to 90% (Fig. 2B).

**TABLE 1:** Sensitivity and specificity of each method for the diagnosis of E. histolytica infection with reference to PCR-confirmed cases

Result	Performance relative to PCR (no. of samples)			Sensitivity (% [95% CI^*a*^])	Specificity (% [95% CI])
	Positive	Negative	Total		
E. histolytica Quik Chek					
Positive	17	1	18	44.7 (30.1–60.3)	
Negative	21	618	639		99.8 (99.1–100)
Total	38	619			
O&P^*b*^					
Positive	25	1	26	65.7 (49.9–78.8)	
Negative	13	618	631		99.8 (99.1–100)
Total	38	619			
Combination of O&P and Quik Chek^*c*^					
Positive	30	2	32	78.9 (63.7–88.9)	
Negative	8	617	625		99.7 (98.8–99.9)
Total	38	619			

a CI, confidence interval.

b O&P, ova and parasite examination.

c Judged as positive when either O&P or Quik Chek produced a positive result.

### Effects of pathogen forms and burden on the sensitivity of the antigen detection test.

**FIG 3:**
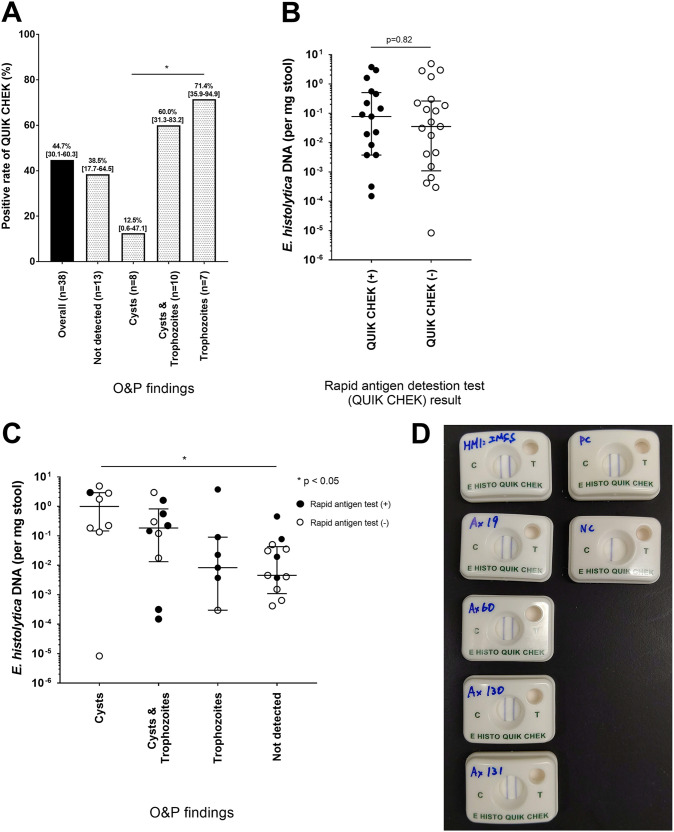
Factors potentially influencing the diagnostic test outcome. (A) The sensitivity of the rapid antigen detection test (Quik Chek) was compared in accordance with the O&P findings. (B) The pathogen burden in the stool samples was determined by quantitative PCR (qPCR) and compared between the positive and negative stool samples based on the antigen detection test. (C) The pathogen burden quantified by qPCR was compared in accordance with the O&P findings. (D) Quik Chek was applied to *in vitro* cultured clinical strains using a concentration of 10^6^ trophozoites/ml. HM-1:IMSS, laboratory reference strain; Ax19, clinical strain from Quik Chek-positive liver abscess sample; Ax60, Ax130, and Ax131, clinical strains from Quik Chek-negative stool samples; PC, positive control; NC, negative control; O&P, ova and parasite examination; Quik Chek, rapid antigen detection test (E. histolytica Quik Chek).

Next, to assess the factors affecting the sensitivity of Quik Chek, we compared the sensitivities of Quik Chek according to the O&P findings. The targeted antigen for this immunochromatography kit is a surface adhesin (Gal/GalNAc lectin) that is highly expressed on the surface of trophozoites (13, 14). As expected, the sensitivity of Quik Chek was only 12.5% for stools containing cysts alone, whereas it was relatively high (70.6%) for trophozoite-containing stools (Fig. 3A). Interestingly, it was shown that Quik Chek identified E. histolytica infection in 38.5% (17.7% to 64.5%) of stool samples with negative O&P results.

The pathogen burden assessed by quantitative PCR for E. histolytica was similar between Quik Chek-positive and -negative stool samples (Fig. 3B). Furthermore, the cyst-containing stools showed a relatively higher pathogen burden than the trophozoite-containing or O&P-negative stool samples (Fig. 3C). We sought to check the potential antigenicity of E. histolytica in the Quik Chek-negative cases by *in vitro* culture. Three strains (Ax60, Ax130, and Ax131) were successfully isolated by *in vitro* passage of the trophozoites from cyst-containing Quik Chek-negative stool samples. Positive Quik Chek results were then obtained using these cultured strains (Fig. 3D). Thus, taken together, the sensitivity of this rapid antigen detection test is more dependent on the form of E. histolytica in the stool samples than on the pathogen burden or genetic properties of each strain.

## DISCUSSION

In the present study, we evaluated the utility of a commercial rapid antigen detection test based on immunochromatography (E. histolytica Quik Chek) in clinical settings in Japan. Notably, Quik Chek showed high specificity (99.8%). On the other hand, the overall sensitivity of Quik Chek in detecting PCR-confirmed cases was 44.7%, although it was shown that Quik Chek exhibited relatively higher sensitivity (60.0%) when looking at diarrheal cases alone. Unexpectedly, in the present study, the sensitivity of Quik Chek was lower than that of O&P, not only for nondiarrheal E. histolytica-positive cases but also diarrheal cases. This was probably because O&P was carried out at core hospitals in Japan by highly trained technicians, resulting in the relatively high sensitivity of O&P. However, in 10 cases, stool samples had to be excluded from O&P analysis because of the unavailability of a technician at the time of sampling. Furthermore, it was shown that 38.5% of the stool samples diagnosed as negative by O&P were diagnosed as positive by Quik Chek. Importantly, it was shown that the combined use of O&P with Quik Chek resulted in increased sensitivity overall. Therefore, Quik Chek could play an important role in the diagnosis of invasive amebiasis in point-of-care settings in Japan.

Here, the sensitivity of Quik Chek was found to be particularly low for cyst-containing stools, which are typically nondiarrheal samples. This was as expected, because the antigen targeted by this assay is an adhesin (Gal/GalNAc lectin) on the surface of the trophozoite form of E. histolytica (11).

The potency of antigen presentation in trophozoite isolates was not assessed in previous studies. In the present study, it was confirmed that Quik Chek showed positive results when using *in vitro* cultured strains derived from cyst-containing antigen-negative stool samples. Thus, the sensitivity of Quik Chek is more dependent on adhesin expression (a phenotypic property of the pathogen) in different stool environments than on the genetic properties of each strain. This indicates that this antigen detection test may be widely applicable to local clinical strains, although more data should be collected from other geographical areas to confirm this. In addition, factors affecting lectin expression in the gut environment, such as the gut microbiome, should be investigated in future studies.

There were several limitations to the present study. First, this study was carried out using anonymized stool samples that were previously submitted for suspected enteric parasitic infections. Hence, the patients’ other clinical data were not available. Factors affecting the sensitivity and specificity, such as antibiotic treatment history and travel history to an area of endemicity, could not be assessed. Moreover, other etiologies of intestinal infectious/noninfectious diseases could not be ruled out. Second, O&P in this study was carried out by highly trained technicians at core hospitals in Japan. The sensitivity and specificity of O&P are highly influenced by technical skill, the level of which differs between health care settings. Local health care settings should, therefore, be taken into consideration before applying our study results.

In conclusion, the present study used a multicenter cross-sectional study design in Japan that confirmed the high specificity of Quik Chek for E. histolytica infection. The combined use of Quik Chek with O&P increased the sensitivity of diagnosis, which may facilitate the detection of *E. histolytic* infection in point-of-care settings in nonendemic situations.

## Supplementary Material

Supplemental file 1
