# Supplementary material for: Utility of the Rapid Antigen Detection Test E. histolytica Quik Chek for the Diagnosis of Entamoeba histolytica Infection in Nonendemic Situations
Source: J Clin Microbiol. 2020 Oct 21;58(11):e01991-20. doi: 10.1128/JCM.01991-20 (PMC7587111; doi:10.1128/JCM.01991-20)
Supplement: Supplemental file 1 [file JCM.01991-20-s0001.pdf]

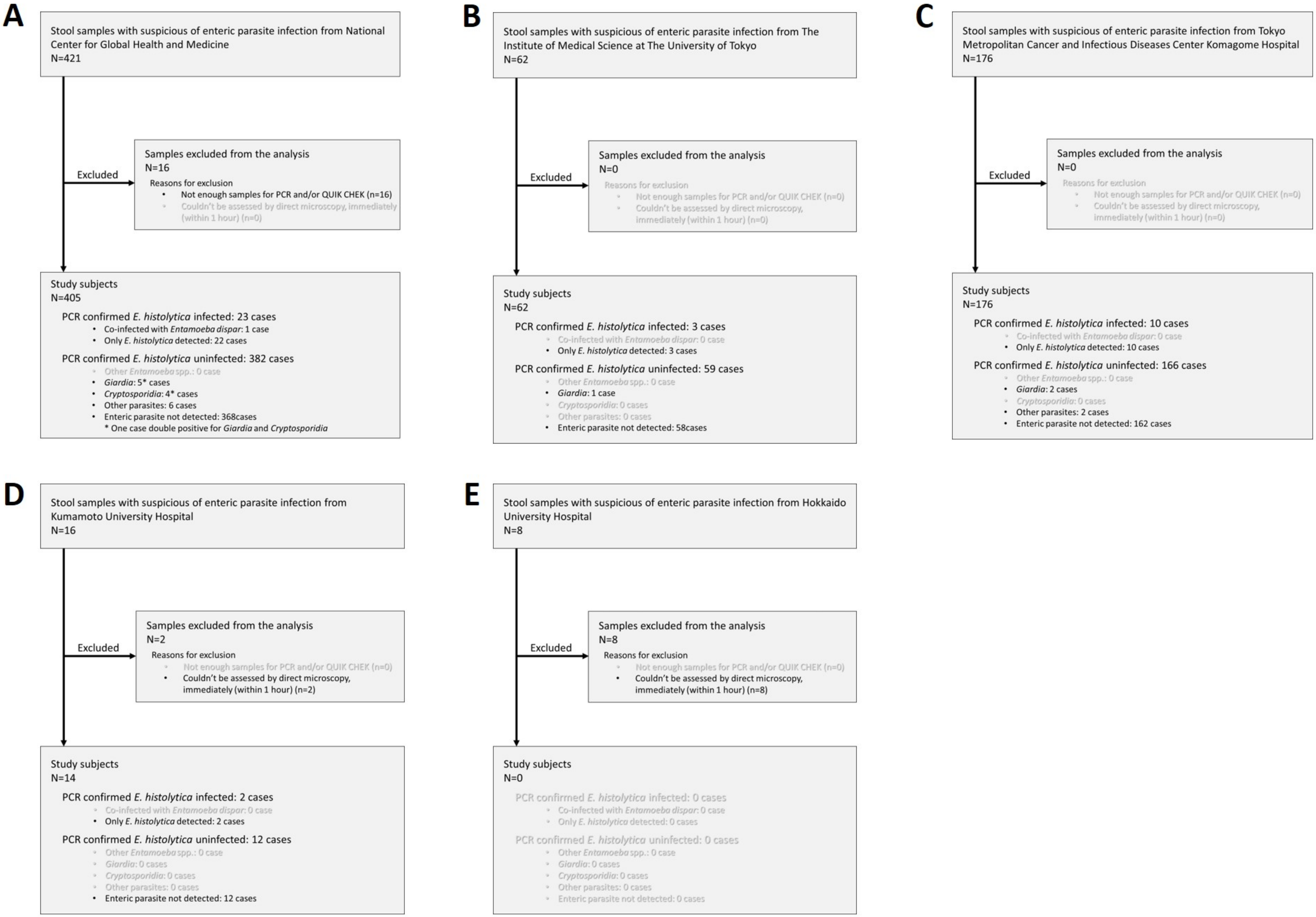

Fig. S1. Workflow of the clinical specimen collection of each institution. (A) National Center for Global Health and Medicine (primary facility, Tokyo), (B) The Institute of Medical Science at The University of Tokyo (Tokyo), (C) Tokyo Metropolitan Cancer and Infectious Diseases Center Komagome Hospital (Tokyo), (D) Kumamoto University Hospital (Kumamoto), and (E) Hokkaido University Hospital (Hokkaido).
